# Sleep to lower elevated blood pressure: study protocol for a randomized controlled trial

**DOI:** 10.1186/1745-6215-15-393

**Published:** 2014-10-09

**Authors:** Emer R McGrath, Colin A Espie, Andrew W Murphy, John Newell, Alice Power, Sarah Madden, Molly Byrne, Martin J O’Donnell

**Affiliations:** HRB Clinical Research Facility, National University of Ireland, Galway, Ireland; Massachusetts General Hospital, Boston, MA USA; University of Oxford, Oxford, UK; National University of Ireland, Galway, Ireland

**Keywords:** Sleep, Hypertension, Cardiovascular disease

## Abstract

**Background:**

Sleep is an essential component of good physical and mental health. Previous studies have reported that poor quality sleep is associated with an increased risk of hypertension and cardiovascular disease. Hypertension is the most common and important risk factor for cardiovascular disease, and even modest reductions in blood pressure can result in significant reductions in the risk of stroke and myocardial infarction. In this trial, we will determine the efficacy of an online sleep intervention in improving blood pressure, in participants with hypertension and poor sleep quality.

**Methods:**

*Trial design:* Randomized-controlled, two-group, parallel, blinded, single-center, Phase II trial of 134 participants. *Population and recruitment*: Primary prevention population of participants with hypertension (systolic blood pressure, 130 to 160 mm Hg; diastolic blood pressure, <110 mm Hg) and poor sleep quality in a community setting. *Intervention:* Multicomponent online sleep intervention consisting of sleep information, sleep hygiene education, and cognitive behavioral therapy. *Comparator:* Standardized cardiovascular risk factor and lifestyle-education session (usual care). *Primary outcome:* Change in mean 24-hour ambulatory systolic blood pressure between baseline and 8-week follow-up. Hypertension has been selected as the primary outcome measure because of its robust association with both poor sleep quality and cardiovascular disease. *Statistical analyses*: Intention-to-treat analysis by using a linear mixed model.

**Trial registration:**

ClinicalTrials.gov: NCT01809821, registered March 8, 2013.

## Background

### Insufficient or poor quality sleep and cardiovascular risk

Sleep is an essential component of good physical and mental health. However, the average sleep duration in the Western world has steadily declined over the past decade [1]. In the National Health Interview Survey 2004 to 2007, more than one third of the North American adult population was noted to have an abnormal duration of sleep, defined as too short (<7 to 8 hours per night) or too long (>8 hours per night) [2]. Previous laboratory and epidemiologic studies showed that inadequate sleep patterns, in terms of both quality and quantity, are associated with an increased frequency of cardiovascular risk factors such as hypertension, diabetes mellitus, and obesity [3, 4], as well as independently associated with an increased risk of adverse cardiovascular outcomes, such as stroke and myocardial infarction [5, 6]. A meta-analysis of 16 prospective cohort studies reported a significant association between sleep of short duration (≤5 to 6 hours per night) and long duration (>8 to 9 hours per night) and an increased risk of all cause-mortality [7]. Therefore, insufficient or poor-quality sleep is a common risk factor for cardiovascular disease (CVD) and all-cause mortality, and represents a potentially important population-based modifiable target for CVD prevention.

### Inadequate sleep and hypertension

A number of epidemiologic studies have reported an association between inadequate sleep, in terms of duration and quality, and an increased risk of hypertension [1, 8–10]. A cross-sectional study among healthy adolescents reported an association between actigraphy-defined low sleep efficiency (an objective measure of sleep quality, defined as the percentage of time in bed estimated to be asleep) and prehypertension, after adjusting for known confounding factors (OR, 3.5; 95% CI, 1.5 to 8.0) [11].

Furthermore, in a substudy of 578 adults from the Coronary Artery Risk Development in Young Adults study, actigraphy-measured shorter sleep duration and lower sleep maintenance (a component of sleep quality defined as the percentage of time between initial sleep onset and final waking that is spent sleeping), were noted in a cross-sectional analysis to be associated with significantly higher systolic and diastolic blood pressures, after adjusting for confounders such as age and antihypertensive medications. In a longitudinal analysis of this cohort, shorter average sleep duration also predicted significantly increased odds of incident hypertension over 5 years (OR, 1.37; 95% CI, 1.05 to 1.78) [12]. In addition, a longitudinal analysis of data from the National Health and Nutrition Examination Survey of >4,500 US adults, reported a significantly increased risk of hypertension in individuals sleeping ≤5 hours per night compared with those sleeping for 7 to 8 hours per night, after adjusting for various potential confounders (HR, 1.32; 95% CI, 1.02 to 1.71) [1].

### Mechanism underlying the association between poor sleep and hypertension

The mechanism underlying the association between insufficient or poor-quality sleep and hypertension is proposed to be multifactorial, including increased sympathetic nervous system activity and increased prevalence of intermediate risk factors, such as poor diet, reduced physical activity, increased weight, and smoking [13]. Laboratory studies have noted significantly increased sympathetic activity and blood pressure in individuals in a sleep-restricted condition, compared with individuals in a sleep-recovery condition [14–17]. Increased urinary excretion of noradrenaline, indicating increased sympathetic activity, has also been reported after a night of sleep deprivation [15, 17]. Increased activity of the sympathetic nervous system can lead to vasoconstriction as well as fluid retention, which can lead to hypertension through volume overload [18].

Blood pressure and heart rate typically exhibit diurnal variation. During sleep, a nocturnal dip occurs in both blood pressure and heart rate, which remain low until the time of awakening. Reduced sleep duration can result in longer exposure to enhanced sympathetic activity and increased average 24-hour blood pressure and heart rate. In this way, habitual sleep restriction can lead to prolonged enhanced sympathetic nervous system activity, the development of hypertension, and subsequently, an increased risk of stroke and other CVD [1].

### Poor sleep and other cardiovascular risk factors

The mechanism underlying the association between poor sleep and other key risk factors for CVD, such as diabetes mellitus and obesity, is thought to be due to sleep-related disruption in metabolic, endocrine, and immune functions. Insufficient sleep has been associated with elevated evening cortisol concentrations, which can result in reduced sensitivity to insulin; increased levels of growth hormone, which can cause a transient insulin resistance; and increased sympathetic nervous system activity, which can inhibit insulin secretion from the pancreas [13, 14, 19]. Thus, sleep-induced disruption of the endocrine and autonomic systems could explain the observed association between inadequate sleep and increased occurrence of diabetes mellitus.

Curtailed sleep has also been associated with reduced levels of leptin, a hormone released by adipocytes, which promotes satiety, and increased levels of ghrelin, a hormone secreted by the stomach and pancreas that stimulates appetite, as well as an increase in subjective feelings of hunger. Thus, this provides a possible mechanism for the observed association between inadequate sleep and obesity [20, 21]. Increased activity of the sympathetic nervous system, with resultant hypertension, is thought to be the underlying pathophysiologic mechanism linking insufficient sleep with diabetes, obesity, and CVD [18]. In turn, these factors also contribute to an increase in blood pressure.

### Current educational interventions to modify traditional cardiovascular risk factors

Currently, stroke and other CVDs are the leading causes of mortality in Europe and North America. Existing interventions to tackle the huge burden of CVD in the population include education on lifestyle modification, as well as the use of primary and secondary preventive therapies [22]. Educational interventions typically consist of advice and information on modifiable risk factors, such as smoking, alcohol consumption, physical activity, and dietary practices. Although factors such as poor diet and lack of physical activity clearly play a central role, another possible contributor to the current epidemic of CVD, mediated through hypertension and the metabolic syndrome, is insufficient or poor-quality sleep [13].

### Modifiability of poor sleep and the potential role of a sleep intervention in CVD prevention

Insufficient or poor sleep quality is a modifiable risk factor for CVD and could potentially be improved through the use of a simple, low-cost, multicomponent intervention aimed at improving sleep quality and quantity. Three previous randomized controlled trials investigating the efficacy of an intervention to improve sleep in participants with insomnia reported significant improvements in sleep efficiency, sleep latency and wakefulness after sleep onset (measures of sleep quality), as well as improvements in mental health and energy [23–25]. Improving sleep quality has been suggested as a therapeutic target for the modification and prevention of cardiovascular risk factors, such as hypertension [1, 12, 26]. Despite its apparent importance, no previous randomized controlled trials have investigated the efficacy of a sleep intervention for lowering blood pressure or reducing the risk of CVD.

In this randomized controlled trial, we evaluate the efficacy of a sleep intervention in controlling blood pressure and other vascular risk factors in a primary prevention population of individuals with hypertension and poor sleep quality.

### Primary aim

To determine whether the addition of a multicomponent, online, sleep intervention to usual care (standard cardiovascular risk factor education), results in a greater reduction in mean 24-hour systolic blood pressure (SBP) in participants with hypertension and poor sleep quality, compared with usual care alone, over an 8-week period.

### Secondary aims

1. To determine whether the addition of a multicomponent, online, sleep intervention to usual care results in a greater reduction in mean 24-hour diastolic blood pressure (DBP) in participants with hypertension and poor sleep quality, compared with usual care alone, over an 8-week period.
2. To determine whether the addition of a multicomponent, online, sleep intervention to usual care results in greater improvements in body mass index (BMI), plasma lipids, plasma HbA1c, estimated glomerular filtration rate (eGFR), mean number of cigarettes smoked per day, Sleep Condition Indicator (SCI) Score, Insomnia Severity Index (ISI) score, Pittsburgh Sleep Quality Index (PSQI) score, sleep-onset latency (SOL), wake time to sleep onset (WASO), sleep efficiency, total sleep time (TST), Beck Depression Inventory (BDI) score and Beck Anxiety Inventory (BAI) score, in participants with hypertension and poor sleep quality, compared with usual care alone, over an 8-week period.
3. To determine whether level of adherence to the sleep intervention influences the change in mean 24-hour SBP over an 8–week period.
4. To determine whether concomitant use of antihypertensive medication influences the change in mean 24-hour SBP over an 8–week period.

## Methods

### Trial design

Randomized, two-group, parallel, blinded, controlled, single-center trial. This is a Phase II, efficacy, proof-of-principle trial with an allocation ratio of 1:1.

### Setting: Identification of participants and description of center

Participants are recruited via advertisements in local newspapers, advertisements on local radio stations, local and online (Twitter and Facebook) poster advertisements, and local public screening events. Interested participants with hypertension (average SBP readings between 130 and 160 mm Hg with average DBP <110 mm Hg) are referred to the Health Research Board Clinical Research Facility Galway (HRB-CRFG) to be screened for trial eligibility. Those who meet the eligibility criteria are then referred to Croí House, Galway City, for baseline risk factor assessment and for provision of written informed consent. Croí House is an established cardiovascular risk factor modification center that provides medical assessment, risk-factor profiling, and educational sessions on cardiovascular risk-factor modification to the local population. Eligible participants are randomized into two management groups: (a) usual care + online sleep intervention, and (b) usual care.

### Choice of study population

Our study is a Phase II trial. A primary prevention population of participants with no previous CVD and poor sleep quality has been chosen because of the high likelihood of such participants being responsive to the intervention and demonstrating a reduction in blood pressure with the intervention. Potential participants already receiving antihypertensive medications are eligible for inclusion in the trial. However, participants with diabetes mellitus and chronic renal impairment are excluded, which should reduce the proportion of participants that will have changes to their blood-pressure medications over the course of the trial.

### Eligibility criteria

#### Inclusion criteria

1. Signed written informed consent
2. ≥ 18 years on entry to study
3. Average automated SBP monitor readings between 130 and 160 mm Hg with average automated DBP monitor readings <110 mm Hg on three occasions, measured in a valid standardized manner while seated; or average 24-hour ambulatory blood pressure monitoring (ABPM) SBP readings between 130 to 160 mm Hg with average DBP readings <110 mm Hg.
4. Self-reported difficulty getting to sleep (defined as usually taking more than 30 minutes to get to sleep), and/or staying asleep (usually waking up more than once per night) for at least 3 months’ duration
5. Internet access, self-reported competency in using the Internet, and regular Internet use

#### Exclusion criteria

1. Currently receiving more than two antihypertensive medications, or recent (within previous 8 weeks) change in type or dose of antihypertensive medications or planned change in antihypertensive medication in next 8 weeks.
2. History of myocardial infarction, ischemic stroke, or transient ischemic attack
3. History of congestive heart failure
4. History of dialysis or evidence of chronic kidney disease (eGFR <60)
5. History of diabetes mellitus
6. Current ongoing sleep-hygiene education or sleep-related cognitive behavioral therapy
7. Ongoing involvement in night-shift work
8. History of obstructive sleep apnea (OSA) and previously received or currently receiving treatment for OSA (participants with a history of untreated OSA are eligible for inclusion).
9. Known history of sleep disorders (that is, narcolepsy; hypersomnias; parasomnias such as sleep walking, night terrors, recurring nightmares; periodic limb movements/restless-leg syndrome; circadian rhythm sleep disorder)
10. Unable to follow educational advice in the opinion of the clinician
11. Baby or young children at home that wake during the night
12. History of bipolar affective disorder
13. History of psychosis
14. History of major depression (defined as depression requiring hospitalization in the past or visit to psychiatry outpatient clinic in the past 3 months)
15. Unstable depression (unstable is defined as changes in antidepressant medications within the last 3 months (that is, starting a new medication, stopping a medication, or changing a medication dose).
16. Unstable anxiety disorders/panic attacks (unstable is defined as changes in anxiolytic medications within the last 3 months (that is, starting a new medication, stopping a medication, or changing a medication dose).
17. Ongoing substance or alcohol abuse
18. Planned surgery or hospitalization during the trial
19. Incapacitating pain or illness or other medical condition in which, in the opinion of the clinician, the sleep intervention is unlikely to be effective.

Participants who are currently taking medications associated with sleep disturbances are not excluded from this study, provided they have been stable on those medications for >3 months (stable is no start, stop, or change in dose in past 3 months). These medications include:

- antidepressants (selective serotonin reuptake inhibitors, venlafaxine, duloxetine, monoamine oxidase inhibitors)
- antipsychotics
- amphetamine derivatives
- decongestants (pseudoephedrine, phenylephrine, phenyl propanolamine)
- narcotic analgesics (oxycodone, codeine, propoxyphene)
- cardiac medications such as β-blockers, α-receptor agonists and antagonists, diuretics, and lipid-lowering agents
- pulmonary medications (theophylline and albuterol)

### Randomization

A randomization strategy using randomly permuted blocks of varying block size is being used, which allows the assignment schedule to be preplanned before participant recruitment. The randomization schedule is constructed by using a computer-generated list of pseudo-random numbers. A centrally administered, computer-generated randomization scheme is being used to assign individual participants randomly in a 1:1 ratio, to either the intervention or control groups. Once a participant is deemed eligible for trial entry and has provided written informed consent, a researcher assigns a unique study-identification number to the participant and contacts the online sleep intervention (Sleepio) team with the participant's name, their assigned participant number, and contact details (Email address and telephone number). The Sleepio team then assigns the participant to the next available randomization number on the list, corresponding to a particular treatment assignment (Sleepio intervention or control). Participants randomized to Sleepio receive a code via Email to access the online sleep intervention. The Sleepio team holds the central record of all participants who have been randomized, along with the corresponding randomization numbers and treatment-group assignments. All investigators at the HRB-CRFG, including the statistician, are blinded to participant treatment-group assignment. At the end of the trial, treatment assignments will be compared with the randomization list, to detect any incorrect assignments, and following completion of statistical analyses, all investigators and trial personnel will be unblinded.

### Blinding

Trial participants, investigators, data collectors, statistician, and outcome assessors are blinded to study-group assignment. Members of the Sleepio team assigning participants to treatment groups are not blinded. Participant consent forms and information leaflets state that a behavioral lifestyle intervention is being compared with a control, and describe the intervention as general education on cardiovascular risk factors. It is also mentioned that aspects of the intervention are delivered via online web sessions, as well as through face-to-face sessions. Cardiology research nurses deliver the usual care intervention (that is, the cardiovascular education intervention) to participants in both treatment arms.

All laboratory measurements are carried out in a blinded fashion. Laboratory samples, with the exception of HbA1c serum levels, are frozen and then stored, and will be analyzed at the end of the trial. Serum HbA1c levels are measured on the day of collection. Blood pressure measurements are recorded in a blinded fashion by using a 24-hour automated blood pressure monitor, thus avoiding observer bias. All other study measurements, including BMI and questionnaire scores, are interpreted and entered into the study database in a blinded fashion by a trained researcher. Because of the nature of the intervention, a requirement for breaking the blinding to access the randomization schedule for a particular participant during the trial is not anticipated.

### Study interventions

#### Components of the sleep intervention

The sleep intervention (Sleepio) is a multicomponent online intervention consisting of sleep information and sleep-hygiene education, along with behavioral and cognitive components. This intervention was developed after consultation with a sleep psychologist. It is based on three previous randomized controlled trials investigating the efficacy of an intervention to improve sleep in participants with insomnia, one of which was an online cognitive behavioral therapy intervention for chronic insomnia disorder [25]. The interventions were similar, with two of the trials showing a significant improvement in sleep efficiency [23, 25], and the third showing a significant improvement in sleep latency, wakefulness after sleep onset, and sleep efficiency (measures of sleep quality), as well as improvements in mental health and energy [24].

Participants in the sleep-intervention arm also receive ”usual” care (standardized cardiovascular risk-factor education program), similar to participants in the control arm. Thus, the comparison is: standardized cardiovascular education + online sleep intervention versus standardized cardiovascular education.

### Multicomponent online sleep intervention

#### Sleep information and sleep-hygiene education

The participant is educated on the need for sleep and its functions, the effects of sleep loss, factors adversely affecting sleep, and the impact of lifestyle habits and environmental factors on sleep. Participants are educated on a series of practical steps to improve sleep hygiene:

- Avoid caffeinated beverages, nicotine, or other stimulants 6 hours before bedtime.
- Avoid alcohol, excessive fluids, and large meals, 3 hours before planned bedtime. Eliminate ”nightcaps”.
- Avoid excessive alcohol intake (14 units for females, 21 units for males, recommend no more than 2 drinks per day)
- Avoid illicit drugs
- Engage in regular physical activity, early in the day.
- Avoid stress and excitement, such as vigorous exercise, in the 2 hours before bedtime
- Avoid intense mental activity requiring a high level of concentration, including computer work, in the 2 hours before planned bedtime.
- Avoid mental activities such as planning or thinking while in bed.
- Maintain an appropriate sleeping environment, in terms of light, noise, ambient temperature, and comfortable mattress/blankets.
- Stimulus control: limit the use of the bedroom to sleeping, with avoidance of nonsleep behaviors such as watching television or reading in the sleep environment. Remove incompatible activities from the bedroom environment (for example, television, books). The aim is to break associations between the sleeping environment and being awake, to re-associate the bedroom with sleep, and to stay in the bedroom only when asleep or feeling sleepy.

#### Sleep scheduling and sleep restriction

To help participants to reshape sleep patterns to correspond with their individual sleep needs. The participant is helped to reestablish a consistent sleep-wake schedule.

- Define individual sleep requirements.
- Develop a good pre-sleep routine
- Establish a strong bed-sleep connection
- Eliminate wakefulness from bed (rising if not asleep within around 20 to 30 minutes)
- Establish and follow a regular and consistent night-to-night sleep schedule, go to sleep in the same bed at the same time each night, and arise immediately on awakening at the same time each morning
- Increase sleep efficiency through scheduling sleep in relation to current total sleep; restrict participants’ allowed time in bed to the actual sleeping time according to the participants’ sleep diary. Adjust the time-in-bed period as sleep efficiency improves from week to week. Participants are instructed to get up at a regular time, even if they did not have enough sleep the night before
- Avoid daytime naps
- Avoid recovery sleep as compensation
- Encourage and support people in changing their sleep routines to improve 7-day per week compliance

#### Cognitive therapy

The participant learns about ways to reduce mental alertness, repetitive thoughts and anxiety that interfere with sleep:

- Identify thought patterns and content that interfere with sleep
- Develop accurate beliefs and attitudes about sleep and sleep loss
- Prepare mentally for bed by putting the day to rest
- Learn thought-distraction and imagery techniques
- Teach progressive relaxation techniques; the participant is taught how to recognize and control muscular tension through use of exercise instructions
- Use these techniques to combat intrusive thoughts
- Reduce efforts to control sleep and allow it to happen naturally
- Encourage and support people in changing their mental approach and to maintain behavior and cognitive change
- Further adjust sleep schedules to maintain sleep efficiency

### Administration of the sleep intervention

Participants receive six weekly sessions delivered by an animated “virtual therapist” (The Prof). These sessions are self-delivered at a convenient time for each participant, over an 8-week period. The program comprises a fully automated media-rich web application, driven dynamically by baseline, adherence, performance, and progress data. At the start of each session, the Prof conducts a progress review with the participant, explores the diary data submitted during the week, the participant’s current sleep status and pattern, and progress achieved against goals previously set. Underlying algorithms feed the delivery of information and provide support and advice in a personally tailored manner. The content of the cognitive behavioral therapy component of the intervention is consistent with the literature, and covers behavioral (for example, sleep restriction, stimulus control) and cognitive (for example, putting the day to rest, thought restructuring, imagery, articulatory suppression, paradoxical intention, mindfulness) strategies, as well as additional relaxation strategies (progressive muscle relaxation and autogenic training) and advice on lifestyle and bedroom factors (sleep hygiene). The intervention is based on a previously validated manual [24, 27]. Table 1 summarizes the content and features of the intervention.

**Table 1:** **Summary of online sleep intervention**

**Treatment content**	Sleep information/education, sleep hygiene, relaxation, stimulus control, sleep restriction, cognitive techniques (restructuring, paradox, mindfulness, imagery, putting day to rest, thought stopping)
**Duration**	Six sessions over a minimum of 6 weeks
**Delivery context**	Fully online
	Delivered by animated therapist (The Prof)
	No face-to-face contact
**Additional treatment features**	Appointment system, interactive sessions, dynamic feedback against personal goals, progress review at start of each session, automatic calculation of sleep data over time, personal case file, end-of-session quiz, 24/7 access
**Support/motivational system**	Praise/reinforcement contingent on progress, online Wikipedia of sleep-educational topics. Social community of users, moderated by experts. Support/prompts/reminders by Email and mobile SMS ”Graduation ceremony” on course completion

#### Methods to maximize participant adherence to the intervention and follow-up procedures

To improve adherence with follow-up procedures, we schedule follow-up visits at a convenient time for participants and ensure that adequate study staff and an efficient system are in place, to minimize waiting times. Participants access an online daily sleep diary throughout the online sleep intervention, which is completed each morning on rising. Participants are able to set automated mobile text message and/or Email prompts as reminders, to further promote adherence. Members of the Sleepio team also contact participants who are not adhering to the intervention by using standardized Emails and phone follow-up, further to encourage adherence to the intervention.

To reduce losses to follow-up, participants who fail to return for a follow-up visit are contacted by phone and/or mail to arrange an alternative convenient appointment.. The cost of transport to and from the study site is reimbursed on request, further to encourage adherence to the follow-up procedures. If participants are unable to make a final follow-up visit to the study site, a home visit with the participant is arranged, where possible, to obtain final outcome data. If the participant refuses or is unable to attend for any further follow-up visits, we attempt to establish the reason for nonattendance. We believe these measures will help ensure a high adherence rate of >85% and a low loss-to-follow-up rate of <5%, thus maximizing the ability of the trial to detect a true treatment effect.

#### Components of the control intervention

The control intervention consists of advice and education on modifying cardiovascular risk factors and adopting a healthier lifestyle. Specific components include advice on smoking cessation, decreasing alcohol intake, increasing physical activity levels, and adopting a healthy diet. This standardized cardiovascular-risk-factor educational session, which represents usual care, is administered in an identical fashion, to trial participants in both arms.

#### Administration of the control intervention

Specialist nurses with specific training in cardiovascular-risk-factor education (working for Croí), administer the cardiovascular-risk-factor education intervention to participants in groups of one to 15 (current average, six to 10), over a 30–minute period. The control intervention is delivered during week 1 of the trial.

### Follow-up schedule

During the initial visit, all eligible participants who provide written informed consent are randomized. Measurements are carried out on two occasions, at baseline and at the final follow-up visit at 8 weeks.

### Measurements

#### Baseline characteristics and measurement of predictor variables

Data are collected at baseline for relevant participant demographic and clinical characteristics. We use programmed portable monitors to obtain ambulatory blood-pressure readings at 30-minute intervals throughout the whole day, and every 60 minutes at night. We define daytime as the interval from 10 AM to 8 PM, and nighttime as the interval from midnight to 6 AM. These fixed intervals eliminate the transition periods in the morning and evening when blood pressure changes rapidly, resulting in daytime and night-time blood pressure levels that are within 1 to 2 mm Hg of the awake and asleep levels. Within individual subjects, we weigh the means of the ambulatory blood pressure by the interval between readings [28].

All vascular risk factors are measured in a standardized fashion, consistent with methods used in INTERHEART and INTERSTROKE studies [29, 30]. Laboratory specimens are collected during baseline and at the 8-week follow-up period. Frozen samples will be analyzed at the GUH laboratory by using standardized storage, handling, and analytic procedures at the end of the trial.

Trained researchers administer the questionnaires (SCI, ISI, PSQI, BDI, and BAI). The data are entered into an electronic case-report form by a trained member of the research team. The following data points are collected:

1. Demographics: date of birth, age, sex, employment status
2. Co-morbidities: history of diabetes mellitus, atrial fibrillation, dialysis, chronic renal disease, chronic kidney disease, congestive heart failure, dyslipidemia, nocturnal asthma attacks, peripheral vascular disease, current smoker (or smoked within last 6 months), mean number of cigarettes smoked per day, alcohol intake, caffeine intake, and level of physical activity
3. Resting heart rate
4. Blood pressure (calibrated, identical machines are used on all participants)
  - Office automated SBP (mm Hg)
  - Office automated DBP (mm Hg)
  - Mean 24-hour ambulatory SBP (mm Hg)
  - Mean 24-hour ambulatory DBP (mm Hg)
  - Daytime peak SBP (mm Hg)
  - Daytime peak DBP (mm Hg)
  - Nighttime peak SBP (mm Hg)
  - Nighttime peak DBP (mm Hg)
  - Mean daytime SBP (mm Hg)
  - Mean daytime DBP (mm Hg)
  - Mean nighttime SBP (mm Hg)
  - Mean nighttime DBP (mm Hg)
5. Current medications: – antihypertensives, statins, other lipid-lowering agents, antithrombotic therapy, oral hypoglycemic agents, insulin, sedatives/hypnotics, and analgesics
6. Sleep questionnaires (PSQI, SCI, ISI)
  - PSQI score
  - PSQI >5
  - Time in bed
  - SOL
  - WASO
  - Total sleeping time (TST)
  - Sleep efficiency
  - SCI score
  - SCI score ≤5.9
  - ISI score
  - ISI score ≥15
7. BAI score
8. BDI score
9. Height, weight, BMI (calculated by using a standard automated weighing scale and a Leicester height measure)
10. Laboratory measurements (nonfasting samples)
  - Plasma lipoproteins: total cholesterol (TC), low-density lipoprotein cholesterol (LDL-C), high-density lipoprotein cholesterol (HDL-C), triglycerides (TGs), apolipoprotein B (ApoB), and apolipoprotein A1 (ApoA1)
  - Plasma HbA1c
  - eGFR

### Study outcomes

#### Primary efficacy outcome

Change in mean 24-hour ambulatory SBP between baseline and 8 weeks of follow-up. Hypertension has been selected as the primary outcome measure because of its robust association with inadequate sleep and its well-documented association with CVD.

#### Secondary outcomes

1. Change in mean 24-hour DBP between baseline and 8 weeks of follow-up.
2. Change in
  - Daytime peak SBP
  - Daytime peak DBP
  - Nighttime peak SBP
  - Nighttime peak DBP
  - Mean daytime SBP
  - Mean daytime DBP
  - Mean nighttime SBP
  - Mean nighttime DBP
3. Change in BMI
4. Change in plasma lipoproteins (TC, LDL-C, HDL-C, TG, ApoB, ApoA1)
5. Change in HbA1c
6. Change in eGFR
7. Change in smoking status
8. Change in mean number of cigarettes smoked per day
9. Change in SCI score
10. Change in ISI score
11. Change in PSQI score
12. Change in SOL
13. Change in WASO
14. Change in total sleep time
15. Change in sleep efficiency
16. Change in proportion of participants with SE ≥85% at 8 weeks
17. Change in proportion of participants with SOL ≤30 minutes at 8 weeks
18. Change in proportion of participants with PSQI <5 at 8 weeks
19. Change in proportion of participants with SCI score ≤5.9 at 8 weeks
20. Change in proportion of participants with ISI score ≥15 at 8 weeks
21. Change in BDI score
22. Change in BAI score

#### Cointerventions

Participants in this trial will ideally not receive any antihypertensive medications throughout the 8-week period. However, participants receiving up to two antihypertensive agents at the time of entry into the trial are eligible for inclusion, provided that no recent (within the previous 8 weeks) changes occurred in type or dose of antihypertensive medications and there are no planned change to antihypertensive medication over the next 8 weeks (during the trial). Note: in patients with mild hypertension and no history of CVD, current guidelines recommend an initial period of lifestyle intervention, consistent with our approach. Treating clinicians will be permitted to use clinical judgment in initiating and dose-adjusting antihypertensive therapies for both study groups during the 8-week trial, where appropriate. Participants who are stable on cardiovascular preventive therapies (no change in previous 3 months), such as lipid-lowering agents, antithrombotic therapies, oral hypoglycemic agents, and insulin will continue their usual treatment. Participants not taking these agents will be encouraged to avoid initiation of treatment with these agents, until the 8-week trial period has been completed. However, treating clinicians will be permitted to use usual clinical judgment in initiating and dose-adjusting such medications, where appropriate. Initiation and/or dose adjusting of cointerventions will be recorded and measured and accounted for using sensitivity analysis. Participant adherence to the intervention will also be measured, and adjusted for using sensitivity analysis. For example, we will analyze the data for evidence of a dose/response relation between degree of reduction in SBP and number of online sleep sessions completed.

### Sample-size calculation

The primary outcome is the mean difference in SBP reduction over the 8-week period between the two study groups. Our estimated minimum clinically meaningful effect size is 5 mm Hg, with an estimated standard deviation (SD) for SBP reduction of 8.3 mm Hg in the intervention group and 7.7 mm Hg in the control group, based on previous research [31]. The minimum effect size is based on previous randomized controlled trials reporting a reduction in CVD with reductions in SBP of 5 mm Hg or greater, and the reduction in blood pressure with lifestyle interventions observed in previous trials [31–33]. A 5.6-mm Hg reduction in blood pressure in the ADVANCE trial resulted in relative-risk reductions of 9% for major vascular events, and 18% for cardiovascular death [34]. Based on a two-sample comparison of means at a significance level of α = 0.05 and power of 80% to detect a difference of 4 mm Hg (power of 90% to detect a difference of 5 mm Hg) between treatment arms, and an estimated 5% loss to follow-up, a per-group sample size of 67 participants will be required.

### Statistical analysis plan

#### Descriptive statistics and baseline characteristics

Descriptive statistics will be used to describe the baseline characteristics of the study sample, the flow of trial participants, and the level of missing data for both predictor and outcome variables. All losses to follow-up and dropouts will be counted, and where possible, reasons documented. Categoric variables will be described by using frequencies and percentages. Histograms and boxplots will be used to evaluate the distribution of continuous variables and to identify any outliers or potential errors in the data, with follow-up verification if necessary from the case-report forms. For continuous variables, the mean and standard deviation will be used to describe the typical value and spread in the sample. For continuous variables that are not normally distributed, the median and interquartile range will be used to describe the typical value and spread in the sample. All tests of significance will be two-sided and conducted at an α = 0.05 for level of statistical significance.

#### Statistical analysis

The primary analysis will use the on-treatment dataset for all outcomes. On-treatment is defined as those participants who completed at least one session of the online sleep intervention. A secondary analysis will use the intention-to-treat dataset, and a third, per-protocol analysis, will also be carried out for all outcomes, with postrandomization exclusions due to ineligibility, noncompliance, loss to follow–up, or missing data.

A linear mixed model will be used to compare changes in mean 24-hour ambulatory SBP (and other secondary outcomes) from baseline to 8 weeks after randomization. We will adjust for covariates associated with the intervention and the outcome as appropriate (for example, age, baseline SBP, baseline DBP, baseline SCI score). The selection of explanatory variables for inclusion in the final model will be based on clinical plausibility and variable selection routines, the LASSO, and regression trees. It will be assumed that missing data are missing at random and therefore accounted for in the mixed model. The validity of this assumption will be investigated by looking at the missing data patterns and by modeling the probability of missing data based on the explanatory variables available. The sensitivity of the missing value-generating mechanism to the choice of variables for inclusion and the final conclusions will be assessed by using multiple imputations via chained equations and predictive mean matching. Model checking will be performed by using suitable model diagnostics and residual plots. All statistical analyses will be performed by using the software packages SPSS version 21.0, R version 2.15.3 and Minitab version 16.2.

#### Subgroup and sensitivity analyses

The primary outcome will be analyzed by subgroups based on

- Age (≥65 years versus <65 years)
- Sex
- Severity of hypertension at baseline (SBP 130 to 150 versus 151 to 160)
- Use of antihypertensive medication at time of entry into the trial
- Introduction of antihypertensive medication during the trial
- Sleep condition indicator score ≤5.9 versus >6.0
- Body mass index (BMI ≤25 versus >25)
- Employment status
- Concomitant use of hypnotics
- Depression, score ≥17 versus <17 on BDI
- Anxiety, score ≥22 versus <22 on BAI
- Adherence to the intervention

Statistical tests of interaction will be performed for all subgroup analyses. A sensitivity analysis will be performed to assess the influence of adherence to the intervention, on the estimated treatment effect. These analyses will use the on-treatment dataset.

### Ethical approval

This study has received ethical approval from the research ethics committee at GUH (ref CA 864).

## Trial management and quality control

### Clinical site

This single-center study will be conducted at Croí House, and the coordinating center will be located at the HRB-CRFG, National University of Ireland, Galway.

### Steering committee and local operations committee

The steering committee will consist of Prof. Martin O’Donnell (Principal investigator), Dr. Emer McGrath (Principal investigator), Mr. Neil Johnson (Croí House), Prof. Andrew Murphy (co-investigator), Dr. John Newell (statistician) and Prof. Colin Espie (University of Glasgow, co-investigator).

### Data collection and quality control

All data collection and all study-outcome measurements are conducted locally. Data are entered into an electronic case-report form by the attending researcher. Data entered include participant identification numbers, verification of eligibility criteria, verification of written informed consent, relevant participant demographic and clinical characteristics, current medications, physical parameters such as blood pressure and BMI, adherence to follow-up, adherence to the intervention, and questionnaire scores. All questionnaire scores are recalculated to ensure validity. Laboratory results for HbA1c, lipoproteins, and eGFR will be automatically entered into the GUH database and will be manually entered into the electronic case-report forms and trial database.

All study personnel are blinded to study-group assignment and will remain blinded until all data have been collated, entered into the database, the full dataset has been blindly reviewed for errors, and statistical analyses have been completed. Blinding will thus help to avoid differential measurement bias or biased data entry.

To ensure that high-quality data are collected, staff receive training on completing case-report forms. In addition, central statistical monitoring of the data by using data-entry edit checks is used, to improve the validity of the data-entry process.

## Discussion

### Methods of minimizing bias

One potential limitation of this study is the risk of contamination, if participants or clinicians become aware of individual treatment-group assignments. This could result in the differential use of co-interventions between the two study groups, or use of a sleep intervention in the control group, resulting in bias toward the null. The screening questionnaire for sleep quality is another potential source of unblinding. To minimize the risk of unblinding, it is being administered as a routine baseline screen, along with questionnaires to measure depression, anxiety, and cognitive function.

A second potential limitation is volunteer bias. Individuals who participate in studies are often more healthy than those who refuse to provide consent. Thus, the actual sample of individuals included in the trial may not be representative of the relevant population, limiting the generalizability of the results. Furthermore, study volunteers are often well-motivated, highly adherent individuals who are likely to benefit from such an intervention. This would also affect the external validity of the results. However, this is a proof-of-concept, phase II study, and such a population will maximize the chances of detecting the true treatment effect, before testing the effectiveness of this intervention in a wider population.

A third potential limitation in this study is the primary outcome measure of reduction in mean 24-hour systolic blood pressure, which is a surrogate outcome measure. As this is a pilot study, a clinical outcome would not be feasible, as a relatively long follow-up period would be required to demonstrate a treatment effect. Reduction in blood pressure has been shown to be predictive of significant reductions in major vascular events. Thus, we believe that reduction of systolic blood pressure is an appropriate outcome measure for this study.

A fourth potential limitation of this study is the risk of nonadherence to the intervention, which is a particular challenge in trials evaluating behavioral interventions. However, a number of measures are being implemented to maximize adherence (see earlier). To determine the influence of adherence on the estimated treatment effect, a sensitivity analysis will be performed.

A fifth potential limitation is that patients with undiagnosed OSA may be included in this study, which could undermine our ability to detect a true treatment effect, given that such patients may not be expected to improve with the intervention. Thus, we have excluded patients with a known diagnosis of treated OSA, to minimize this potential limitation. Finally, our results could be subject to attention bias, due to differences in the level of attention provided to the two treatment groups. However, given that this is a phase II, proof-of-concept study, with the aim of maximizing the chances of detecting a true treatment effect, we elected not to use an attention placebo in the control arm. If a significant treatment effect is observed in this pilot study, a larger-scale phase III trial will be performed, with the inclusion of an attention placebo in the control arm, to demonstrate the effectiveness of this intervention in a broader population of patients for primary and secondary prevention of CVD.

## Trial status

Ongoing, participant recruitment under way.

## Abbreviations

ApoA1: Apolipoprotein A1
Apo B: Apolipoprotein B
BAI: Beck Anxiety Inventory
BDI: Beck Depression Inventory
BMI: Body mass index
CVD: Cardiovascular disease
DBP: Diastolic blood pressure
eGFR: Estimated glomerular filtration rate
GUH: Galway University Hospital
HRB-CRFG: Health Research Board-Clinical Research Facility Galway
ISI: Insomnia Severity Index
HDL-C: High-density lipoprotein cholesterol
LDL-C: Low-density lipoprotein cholesterol
OSA: Obstructive sleep apnea
PSQI: Pittsburgh Sleep Quality Index
SOL: Sleep-onset latency
SBP: Systolic blood pressure
SCI: Sleep condition indicator
SD: Standard deviation. TC, total cholesterol
TG: Triglyceride
TST: Total sleep time
WASO: Wake time to sleep onset.
